# SKELETAL MUSCLE ADIPOSITY IS ASSOCIATED WITH LOWER COGNITION IN AFRICAN CARIBBEAN WOMEN

**DOI:** 10.1093/geroni/igac059.2275

**Published:** 2022-12-20

**Authors:** Adrianna Acevedo-Fontanez, Ryan Cvejkus, Allison Kuipers, Joseph Zmuda, Caterina Rosano, Iva Miljkovic

**Affiliations:** University of Pittsburgh, Pittsburgh, Pennsylvania, United States; University of Pittsburgh, Pittsburgh, Pennsylvania, United States; University of Pittsburgh, Pittsburgh, Pennsylvania, United States; University of Pittsburgh, Pittsburgh, Pennsylvania, United States; University of Pittsburgh, Pittsburgh, Pennsylvania, United States; University of Pittsburgh, Pittsburgh, Pennsylvania, United States

## Abstract

**Objective:**

Skeletal muscle adiposity (SMA) increases with aging and is recognized as a major risk factor for cardiometabolic diseases, disability, and mortality among older adults. Obesity is related to dementia and cognitive decline yet the relationship between SMA and cognition remains ill defined. The objective of this study was to assess SMA and cognitive function among African Caribbean women.Design and

**Methods:**

Cross-sectional analysis of 448 African Caribbean women in the Tobago Health Study (mean age, 55 years; range, 39-84 years). Cognition was assessed by the Digit Symbol Substitution Test (DSST), a test of information processing speed with a range of 0-90; higher scores suggest better cognitive function (faster information processing speed). Calf SMA (muscle density) was assessed with computed tomography (Stratec XCT-2000). Linear regression was used to assess the association of SMA with DSST adjusted for age, education, muscle area, waist circumference, alcohol intake, smoking, physical activity, diabetes, and hypertension.

**Results:**

Participants had a BMI of 30.7 kg/m2. Mean (SD) DSST scores and SMA were 39.2 (13.1) and 71.7 (5.3) mg/cm3, respectively. After full adjustment, we found that one SD greater skeletal muscle adiposity was associated with a 1.40 lower DSST score (p-value=0.025).

**Conclusions:**

Our findings suggest that in African Caribbean women, greater SMA is associated with slower information processing speed, an early indicator of future dementia risk. Future studies using an expanded battery of cognitive tests and longitudinal follow-up should further advance our understanding of the role of SMA and dementia risk among African ancestry populations.

